# Alzheimer's Disease Clinical and Research Update for Health Care Practitioners

**DOI:** 10.1155/2013/207178

**Published:** 2013-09-04

**Authors:** Philip A. DeFina, Rosemarie Scolaro Moser, Megan Glenn, Jonathan D. Lichtenstein, Jonathan Fellus

**Affiliations:** ^1^International Brain Research Foundation Inc., 227 Route 206 North, Building 2, Suite 101, Flanders, NJ 07836, USA; ^2^RSM Psychology Center, LLC 3131 Princeton Pike, Building 5, Suite 110, Lawrenceville, NJ 08648, USA

## Abstract

Of the approximately 6.8 million Americans who have been diagnosed with dementia, over 5 million have been diagnosed with Alzheimer's Disease (AD). Due to the rise in the aging population, these figures are expected to double by 2050. The following paper provides an up-to-date review of clinical issues and relevant research. Research related to the methods of the earliest possible detection of AD is ongoing. Health care professionals should play a critical role in differentially diagnosing AD patients, as well as supporting their families. Novel interventions, including medications, natural supplements, and behavioral techniques, are constantly appearing in the literature. It is necessary for the health practitioner to remain current, regarding AD, as such information will facilitate better care for patients and their families.

## 1. Introduction

Approximately 6.8 million individuals in the United States are affected by dementia [1], and approximately 5.3 million have been diagnosed with Alzheimer's Disease (AD) [2]. As the elderly population continues to grow, these numbers are only expected to increase. AD has emerged as a serious public health concern, placing an immense burden on the individual, family, community, and health care resources.

AD most frequently presents with episodic memory impairment as the earliest and most prominent feature, with additional deficits in language, semantic memory, executive functioning, visuospatial abilities, and functional impairment that emerge over the disease course [3]. A common misconception is that AD is a “normal” or expected occurrence of aging, and it is part of the typical trajectory of age-related cognitive decline. Rather, healthy aging has been found to be associated with relatively stable performance on measures of cognitive functioning when measured longitudinally. However, cross-sectional studies have indicated that some domains of cognitive functioning do in fact decline with age [4]. As individuals live to advanced ages (e.g., over the age of 80), it can become more challenging to differentiate between the subtle cognitive declines that accompany aging and those that signify early dementia [5].

The trajectory of AD is characterized along a continuum, ranging from healthy aging to preclinical AD, mild cognitive impairment (MCI), and dementia. Pathological changes that underlie AD begin to accumulate for years, or even decades, before emotional, physical, or cognitive symptoms emerge, eventually reaching a threshold at which the onset of a gradual and progressive decline in cognition occurs [5]. Preclinical AD constitutes the presymptomatic phase during which characteristic neuropathological changes begin to emerge [6].

The transitional period between normal cognitive functioning and dementia is referred to as Mild Cognitive Impairment (MCI) [7]; the most common form, the one most likely to progress to AD, is amnestic MCI (a-MCI). Patients with a-MCI present with memory deficits greater than would be expected based on age and education; however functional abilities remain relatively preserved and independence intact [8]. Estimates of the annual incidence of individuals with MCI progressing to dementia range from 5 to 15% [9]. Regardless of this variation, annual conversion rate of those with MCI is far greater than the baseline incidence rate [4].

AD is often referred to as a “family disease” because of the tremendous impact that befalls the patient's immediate social support system. By identifying AD in its early stages, recommendations for the most current or efficacious interventions can be made, with the goal of slowing disease progression. Early detection may provide patients and their families with an opportunity to begin the discussion of future caregiving, finances, and end-of-life issues before the patient's autonomous decision-making skills deteriorate. Also, implementing caregiver interventions, such as referral to support groups, psychoeducation, and counseling or psychotherapy [9], can also assist patients and their families.

## 2. Epidemiology and Pathogenesis 

Over the past 20 years, researchers have made great strides in the areas of AD, with respect to etiology, prevention, diagnosis, and treatment. However, while the exact etiology still remains a mystery, definitive diagnosis can only be made postmortem, and current treatments can only slow disease progression temporarily. Late-onset AD, the most common form of the disease, occurs in individuals over the age of 65. While researchers have not found any causal determinants for this particular type, they have identified several associated risk factors, including age, female gender, low educational and occupational attainment, prior head injury, sleep disorders (e.g., sleep apnea), estrogen replacement therapy, and vascular risk factors, such as diabetes, hypercholesterolemia, and hypertension [5, 10]. Additionally, the apolipoprotein E (APOE) gene has been recognized as conferring an increased likelihood of developing late-onset AD. Depending on the combination of APOE alleles that an individual possesses, he or she may have a three to eight times higher risk. The much rarer, early-onset from of AD, occurring in fewer than five percent of individuals with the disease, typically affects individuals between the ages of approximately thirty and sixty. This form of the disease is caused by one of three identified genetic mutations that are passed down in an autosomal dominant fashion among families: the amyloid precursor protein (APP), presenilin-1, and presenilin-2 genes [4, 10].

AD is characterized by progressive degenerative neuronal changes, with associated global deterioration of cognitive and personality functioning. This pathological sequence preferentially begins in the medial temporal lobe structures responsible for memory (the entorhinal cortex and hippocampus) and then progresses to the frontal, temporal, and parietal areas, with relative sparing of the motor and sensory cortical regions and subcortical regions [11]. The most widely held theory accounting for the pathological changes underlying disease process is the amyloid cascade hypothesis, positing that the primary, triggering event is the excessive accumulation and clumping together of beta-amyloid (A*β*), leading to the formation and deposition of amyloid plaques throughout the medial temporal lobe and cerebral cortex [12]. A resultant “cascade of events” occurs, including neuronal damage (and eventually death), disrupted neuronal communication, inflammation, and the initiation of a second abnormal protein process—the accumulation of neurofibrillary tangles (NFTs) [4, 13].

NFTs are composed of an abnormal form of the intraneuronal protein tau, which normally plays a role in structural support and cellular communication. Abnormal processes cause the tau protein to “misfold” and aggregate into NFTs, ultimately leading to a breakdown in neuronal function and communication and eventually cell death [14]. The accumulation of NFTs occurs in a hierarchical pattern, beginning primarily in the medial temporal lobe (especially, the entorhinal cortex), gradually progressing into the limbic system (hippocampus and amygdala), and eventually spreading throughout the neocortex [4, 11, 15]. There is evidence that the presence of both amyloid plaques and NFTs is required for AD to develop [8].

Researchers continue to search for tools that can offer the same degree of diagnostic certainty during life that postmortem brain tissue examinations offer. There are currently five biomarkers which show the most promise as indicators of AD pathology organized into two categories: biomarkers of beta-amyloid accumulation and biomarkers of neuronal degeneration or injury [16]. The accumulation of beta-amyloid can be detected through the use of radioactive tracers in conjunction with positron emission tomography (PET) imaging [17], as well as through the analysis of beta-amyloid levels in the cerebrospinal fluid (CSF) [18]. Analysis of CSF levels of tau has also been found to indicate neuronal degeneration associated with NFT accumulation [8, 18]. Fluorodeoxyglucose- (FDG-) PET imaging can be employed to detect hypometabolism in the temporoparietal region, which has been shown to effectively differentiate AD from normal controls [19]. Finally, structural magnetic resonance imaging (MRI) can be used to detect the characteristic pattern of pronounced atrophy in the medial temporal lobes that often occurs in mild to moderate AD [20].

While biomarker research holds promise for early detection and diagnosis of AD, standardized guidelines are still being developed for determining cut-points for diagnosis [8]. Thus, the use of biomarker data is currently indicated primarily for research purposes. Newly approved amyloid imaging techniques (via PET scan) are beginning to be used in order to supplement the results of other diagnostic evaluations.

## 3. Clinical Manifestations

In 2009, the National Institute on Aging and the Alzheimer's Association (NIA-AA) joined forces to develop new up-to-date guidelines for diagnosing AD based on the most current state of the evidence regarding the clinical and pathological processes of the disease. The official criteria were published in 2011 and are summarized as follows: (1) a gradual, progressive decline in cognition that represents a deterioration from a previous higher level; (2) cognitive or behavioral impairment evident in at least two of the following domains: episodic memory, executive functioning, visuospatial abilities, language functions, personality and/or behavior; (3) significant functional impairment that affects the individual's ability to carry out daily living activities; (4) the symptoms are not better accounted for by delirium or another mental disorder, stroke, another dementing condition (i.e., vascular dementia, frontotemporal dementia) or other neurological condition, or the effects of a medication [16]. The Diagnostic and Statistical Manual of Mental Disorders is a tool which is widely employed in clinical settings for diagnosing AD. The recently released version, the DSM-5, contains updated criteria for diagnosing AD which parallel the NIA-AA diagnostic guidelines. It is imperative for clinicians to familiarize themselves with these revised criteria, listed within the Neurocognitive Disorders section, as the criteria contained in the prior DSM-IV-TR are not reflective of the current state of the AD literature [21].

The initial presentation of AD typically involves anterograde amnesia resulting from progressive declines in episodic memory. Specific memory tests may reveal deficits in the encoding and consolidating of new information into long term memory as evidenced by rapid forgetting after a time delay and lack of improvement even when recognition cues are provided. On episodic memory tasks, AD patients commonly commit more errors of intrusion and perseveration, have difficulty employing semantic encoding tactics, and demonstrate less of a primacy effect when compared to normal elderly individuals [3, 5, 23]. As memory impairment begets functional decline, some of the first overt signs of AD often noted by family members include repeating oneself in conversations, misplacing items, becoming lost while driving (familiar routes), burning meals while cooking, and difficulty managing finances [4, 24]. With regard to remote memory, a pattern emerges in the early stages of the disease in which older memories are relatively spared, while those from the more recent past are lost [5, 23].

Deficits in semantic memory and language may become evident early in the course of AD, as well. These difficulties are thought to result from the degenerative disease process causing a breakdown in the brain's interconnected network of general knowledge for concepts, facts, words, and their meaning. Impairment may be detected on tests of verbal fluency, with the tendency to perform relatively worse on tasks requiring generation of words from a given category versus generation of words that begin with a particular letter of the alphabet. Patients are unable to employ clustering strategies to boost their performances and are also unaided by category retrieval cues [3, 23]. Given that AD leads to a loss of semantic knowledge, the failure to demonstrate semantic knowledge for a particular item or concept has been shown to be consistent across test methods [3]. Poor performance is also typically seen on confrontation naming tests and semantic categorization (i.e., of pictures) [5, 23]. Language discourse becomes increasingly filled with circumlocutions and overlearned phrases, accompanied by diminished meaning and spontaneity [23]. Decline in executive functioning can be seen on tests of complex problem solving, working memory, mental flexibility, and sequencing; deficits in these areas may be detected relatively early on tests such as the Tower of London puzzle, Porteus Maze task, Trail-Making Test, and Wisconsin Card Sorting Task and are implicated in the decline of instrumental activities of daily living (IADLs). Tests of immediate attention span and focus, such as digit span and mental control, may remain intact until later in the disease progression [3, 5, 23].

Visuospatial functioning tends not to be a prominent early feature of AD, but instead it regresses over the course of the disease [5]. In particular, visuoconstructional deficits may be apparent on the Clock Drawing task and on complex copying tasks using drawing or blocks [23, 24]. A hallmark indicator of AD is the patients' tendency to perform their copy of a design extremely close to, touching, or on top of the stimulus item [23]. Additionally, visuoperceptual and visual orientation abilities may become disturbed over time [5].

While extrapyramidal motor signs are more prominent in the latter stages of AD, patients may show deficits in ideomotor (skilled movement to verbal command or imitation) and ideational (performing a planned series of motor tasks to achieve a goal) praxis, even in the early stages of the disease [24]. This has implications for their ability to independently perform daily living tasks [23].

## 4. Diagnostic Approach

Determining the primary cause of cognitive decline can be challenging, given the common comorbidity of cerebrovascular disease, Lewy body disease, and AD [6, 8]. Additionally, temporal conditions, such as delirium, depression, anxiety, metabolic disorders, vitamin deficiencies, normal pressure hydrocephalus (NPH), and adverse reactions to medication, may resemble AD particularly in individuals who are young, newly symptomatic or whose symptoms are mild [24]. Neuropsychological testing can contribute to differentiating a true dementia process from a pseudo-dementia, as well as distinguishing between different forms of dementia. However, there are times when patients meet the diagnostic criteria for AD, and have a comorbid condition that may be contributing to cognitive impairment, thus causing a mixed dementia. The NIA-AA criteria differentiate between probable and possible AD and designate patients with mixed dementias to the latter category [16].

Table 1 offers a schematic for diagnostic guidance when evaluating a patient for dementia. Generally, when a clinician suspects changes in mental status, a screening test is performed to assess global cognitive abilities. The Mini-Mental State Examination (MMSE) is among the most widely researched instruments for screening cognitive impairment. This global assessment tool measures orientation to time and place, word recall, language abilities, attention and calculation, and visuospatial skills. The MMSE yields a perfect score of 30, with cut-off points between 24 and 26 suggesting dementia [25]. Age and education adjustments should be utilized for MMSE scoring, as performance may be affected by demographic factors, particularly education [25]. Temporal orientation and factual understanding of current events may be problematic even in early AD, which may be revealed by the MMSE.

The Montreal Cognitive Assessment (MoCA) was more recently developed to specifically detect the more subtle deficits associated with MCI [26]. Similar to the MMSE, scores on the MoCA range from 0 to 30, with the suggested cut-off for impairment being less than 26. However, the MoCA includes more difficult tasks, making it more sensitive in differentiating normal cognition from mild cognitive impairment. Individuals with MCI often score in the normal range on the MMSE and in the impaired range on the MoCA, highlighting the utility of the MoCA in detecting the earliest symptoms of AD [26].

A thorough patient history should be taken, preferably involving a knowledgeable spouse or other family member, in order to determine whether the onset and course of cognitive decline are gradual and progressive, as in AD, or sudden and/or stepwise, which may occur in the case of vascular dementia. An appropriate set of medical tests (i.e., neuroimaging, laboratory tests) may be conducted to rule out another neurological condition, medical illness, or another process which may be present, and patients are often referred for a neuropsychological assessment [8, 16]. 

The neuropsychologist should utilize a battery of assessment measures that are sensitive to the cognitive deficits seen in AD and capable of distinguishing between age-related cognitive decline, MCI, AD, and other forms of dementia. An example of such an abbreviated battery, used by the Alzheimer's Disease Centers (ADC) program of the NIA, incorporates measures of global cognition (MMSE), attention (Digit Span Forward & Backward), processing speed (Digit Symbol; Trail Making Test—Part A), executive functions (Trail Making Test—Part B), episodic memory (Logical Memory Story A—Immediate and Delayed Recall), and language (Category Fluency; Boston Naming) [27]. Additional evidence of episodic memory impairment may be gathered from word list memory tasks, which can help identify deficits in encoding, storage, and retrieval. Combined visuoconstructional and visual memory tasks may be used which require the patient to copy shapes and then to recall those shapes after a delay, both from memory and with recognition cues. A diverse range of skills may be assessed by administering a Clock Drawing task, including planning, visual attention, spatial orientation, and graphomotor control [28]. Finally, since many patients with dementia have never undergone previous neuropsychological assessment, an estimate of premorbid IQ may be obtained by administering a word list reading task, such as the Wechsler Test of Adult Reading (WTAR).

In addition to evaluating the patient's cognitive, the neuropsychologist will address functional issues related to the patient's personal safety and ability to perform instrumental activities of daily living (IADLs), such as driving, medication management, and financial management. Ability to perform basic activities of daily living (BADLs) must also be examined (e.g., toileting, bathing, and dressing), through observation, informant report, or rehabilitation specialists such as occupational therapists. The “gold standard” of formal measures for assessing the level of functional impairment is the Clinical Dementia Rating Scale (CDR) [29], which is a semistructured interview combining information from the patient and a knowledgeable informant. A global rating of dementia severity is calculated, along with the CDR sum of boxes score (CDR-SB), which allows for a detailed, continuous measure of the subtle differences between levels of impairment and has been found to be able to discriminate MCI from early AD [30].

## 5. Therapeutic Interventions

In the management of AD, a multimodal approach is warranted.  Table 2 provides an outline of eight intervention areas to address. These interventions include pharmaceutical, nutraceutical, medical foods, neurophysiological, physical health, cognitive, behavioral, and future planning. 

At this time, there are no established therapeutic interventions that have been found which can stop the progression or reverse the neural deterioration caused by AD. However, there are four FDA approved pharmaceuticals currently prescribed which temporarily halt or slow cognitive, functional, and behavioral decline. Three of the medications are cholinesterase inhibitors, namely, Donepezil, Rivastigmine, and Galantamine, which work to increase the levels of acetylcholine, a neurotransmitter in the brain that is involved in learning and memory. The cholinesterase inhibitors are indicated for the treatment of individuals in the mild to moderate stages of AD [31, 32]. Memantine works by increasing the levels of glutamate, another neurotransmitter implicated in learning and memory. This drug is indicated for the treatment of moderate to severe AD. There is also evidence that Memantine may provide added benefits for individuals with AD who are already taking Donepezil [33]. Overall, the benefits of AD drugs are limited, as they are effective for approximately one year and in only about half of individuals to whom they are prescribed [31].

Currently, there are no other evidence-supported treatments for AD, however ongoing research aims to find disease-modifying treatments. Consensus statements have pointed to a multifaceted approach for conquering AD, using a combination of drugs to target a number of factors associated with the disease process, including A*β* deposits, NFTs, inflammation, immune dysregulation, and insulin resistance [34]. Recent breakthroughs include results from a phase II clinical trial of IVIG, an immunotherapy agent, which was found to stabilize cognition and functioning, in a small sample of AD patients, for three years [35]. Another promising finding came from a pilot clinical trial of an intranasal insulin therapy for AD and a-MCI in which participants who underwent treatment experienced memory improvement and/or maintained their current level of overall cognitive and functional performance [36].

Additionally, Alpha GPC, phosphatidylserine, Huperzine A, and choline show promise as nutraceutical agents for enhancing cognitive performance and slowing cognitive decline. Alpha GPC, also known as L-Alpha Glycerylphosphorylcholine, a naturally occurring form of choline, acts as a parasympathomimetic acetylcholine precursor and has shown promise in improving cognitive symptoms related to AD, vascular dementia, and multi-infarct dementia. Phosphatidylserine is a widely abundant anionic phospholipid in the human body and has been shown to improve age-related cognitive changes. Huperzine A (a natural cholinesterase inhibitor) has been linked to improved memory performance in elderly people with benign forgetfulness, as well as patients with AD and vascular dementia. Cholinesterase inhibitors have been shown to have neuroprotective properties in patients with mild [37] as well as moderate-to-advanced AD [38].

Recently, there is the development of medical foods that are thought to have some promise in improving mental status: Axona, CerefolinNAC, and Souvenaid [39]. Each works via a different mechanism of action, and all are prescriptive supplements. However, Souvenaid is not currently available for use in the USA. 

The application of translational models, such as through animal and cell research, has helped identify certain processes and elements that may deter the neuropathogenetic progression of AD [40]. Research has begun to explore nonpharmaceutical interventions through translational models that may reduce toxins and prevent cell loss including apoptosis. In other words, once applications of interventions on animals or cells are deemed successful, they can be translated or applied to human participants. Laser light therapy is one such intervention, and animal studies using infrared light treatment have documented positive results in mice with traumatic brain injury [41]. Stimulation of human mitochondrial processes and cell proliferation due to laser irradiation have also been demonstrated [42]. More recently, researchers revealed a significant reduction of Amyloid-B aggregates in neuroblastoma cells that were irradiated with intense 670 nm laser light, leading the authors to suggest that their approach might inspire a practical therapy for AD [43].

Ultimately, the most successful model of treatment for AD will likely include early detection and control of physical factors (diabetes, hypertension, hyperlipidemia), followed by application of multifaceted, disease-modifying interventions to prevent the early and continued loss of neurons and to reduce the toxins that result in further cell deterioration [34].

Changes in personality and behavioral disturbances affect most patients with AD and can range from disinterest and apathy to agitation, affective disinhibition, and restlessness. Specific behaviors can be difficult to manage, such as aimless wandering, emotional outbursts, stubbornness, paranoia, hallucinations, and depression. Behavioral interventions can complement medication management and include creating a structured, safe, low stress environment, promoting regular sleep and eating habits, minimizing unexpected changes, and employing redirection and distraction [44].

Since ADLs such as self-care, personal hygiene, and dressing tend to worsen with the progression of the disease, patients with advanced AD require a greater level of caretaker commitment. Caregivers should be alerted to the challenges they will face as the disease progresses and be provided with appropriate coping skills, training, and interventions, through support groups and individual therapy. When at-home care is no longer an option, families will face the decision of placing their loved one in an assisted-living facility. Caregivers should not make this choice in isolation; mental health practitioners can help provide information and allow for the processing of the emotional weight of the decision and any mixed emotions of guilt, hurt, anger, and loss.

Considering that IADLs also decrease in AD, issues such as management of medical decisions, financial affairs, and cessation of driving will also emerge. When the patient is no longer able to perform basic math calculations, securing a financial advisor to oversee assets is often recommended. When insight becomes limited and memory is significantly compromised, medical decision-making and medication management may also need to be shifted to the hands of a caregiver. Pursuit of guardianship and capacity evaluations are not uncommon, especially when estate and legal issues need to be addressed.

## 6. Preventative Interventions

AD is believed to emerge as the result of a complicated interplay of genetic, environmental, and lifestyle factors. Due to this complex process, it is difficult to pinpoint a definitive prevention strategy; however, there is mounting evidence that modifying certain lifestyle factors may lower the risk of developing AD [45]. There is data to suggest that aerobic exercise may improve cognition [5] and serve a protective role in healthy older adults by inducing neuroplasticity in areas of the brain associated with episodic memory [46]. Additionally, physical activity has been found to improve scores on cognitive and functional measures in individuals with MCI and dementia [47].

As cardiovascular risk factors, such as diabetes, hypercholesterolemia, and hypertension, have been found to be associated with AD, it is hypothesized that preventing or managing these conditions may decrease the likelihood of developing AD [45]. Research has shown that healthy eating, specifically adhering to a Mediterranean diet, correlated with both a lower risk of cardiovascular disease and AD [48]. While there is ongoing research investigating the effects of various vitamins and dietary supplements in preventing AD, as of yet, clinical trials have not been able to prove their effectiveness [45].

In addition to maintaining physical health, engagement in cognitively stimulating as well as social activities seems important for promoting healthy brain functioning. Investigators have found that older adults who frequently participate in mentally demanding activities (i.e., reading, crossword puzzles) have decreased odds of developing AD [49]. Formal interventions involving cognitive training and time spent engaging in physical, cognitive, and social activities have been associated with a lower risk of developing dementia in healthy older adults, especially for individuals who participated in two or three of these endeavors [50].

## 7. Summary

Alzheimer's Disease (AD) is an increasingly common condition with projected increased incidence rates in the population. Fortunately, research geared towards enhancing disease-modifying and preventative interventions is gaining momentum. Neuropsychological evaluation continues to play a critical role in early detection and differential diagnosis of normal aging versus MCI and the various types of dementia. Health care practitioners can offer strategies and support for patients, as well as their families and caregivers, related to the disruptions that AD has upon daily functioning. As researchers continue to make strides in our understanding of the disease, it is imperative for clinicians to remain abreast of the dementia literature in order to assist patients in obtaining the most effective care.

## Figures and Tables

**Table 1:** Diagnostic assessment for dementia.

(i) Initial exam
(signs to look for)
Poor orientation
Increased forgetfulness
Confusion in ADLs and IADLs
Language perseverations and circumlocutions
Change in personality and emotional status
Avoidance of typical activities and hobbies
Social isolation
(ii) Mental status screening-use NIAA criteria
(example of evaluation tools that may be used)
Mini mental state
Clock drawing
Mattis dementia screening
Montreal cognitive assessment
Clinical dementia rating scale
Geriatric depression scale
(iii) Interview for instrumental activities of daily living
(observations by family or caregiver report)
Medication use
Cooking
Driving
Financial management
(iv) Interview for general activities of daily living
(observations by family or caregiver report)
Bathing
Dressing
Toileting
(v) Assessment of visual motor skills
(signs to look for)
Ideomotor apraxia (skilled movement to verbal
command or initiation)
Ideational apraxia (performing a planned series
of tasks to achieve a goal)
Extrapyramidal motor signs
Constructional apraxia
Spatial conceptualization errors
(vi) Neuroimaging
CT
SPECT
MRI
PET
(vii) Neuropsychological testing
(skill areas to assess)
Estimate of premorbid IQ
Attention
Processing speed
Executive functioning
Planning, organization, mental flexibility
Memory
Working memory
Immediate recall
Delayed recall
Long term memory
Language
Naming
Semantic fluency
Evidence of perseverations
Evidence of circumlocution

**Table 2:** Management of dementia: eight intervention areas to be addressed.

(i) Pharmaceutical interventions	
(medications that may provide symptom relief for cognitive, emotional, and behavioral issues)	
Donepezil	
Rivastigmine	
Galantamine	
Memantine	
Psychotropic (antidepressants, antianxiety, mood stabilizer, antipsychotic)	
(ii) Nutraceutical interventions	
(dietary supplements still in research stages)	
Omega 3	
Curcumin	
Vitamin D	
Alpha GPC	
Phosphatidylserine	
Choline	
(iii) Medical foods interventions	
(prescriptive)	
Axona	
CerefolinNAC	
Souvenaid (not currently available in the USA)	
(iv) Neurophysiological interventions	
(still in research stages)	
Cranioelectric stimulation	
Transcranial magnetic stimulation	
Neurofeedback	
Laser light therapy	
(v) Physical health interventions	
Aerobic exercise	
Medical management of disease	
Diabetes	
Hypertension	
Hyperlipidemia	
(vi) Cognitive interventions	
Mental exercises/hobbies	
Compensatory memory strategies and aids	
Cognitive training	
(vii) Behavioral interventions	
(strategies to manage factors such as wandering, sundowning, agitation, disorientation, affective disinhibition, stubbornness, paranoia, irritability, apathy, and restlessness)	
Occupational therapy safety assessment of home	
Low stress calming environment	
Regular sleep and meal schedules	
Redirection and distraction	
Minimization of unexpected changes in environment	
Patient counseling and support groups in early stages	
Caregiver strategies and resources through support groups and internet	
In-home aide/assistant	
Outpatient day program with all inclusive care	
assisted living facility	
(viii) Future planning interventions	
Caregiver counseling to aid in life planning and decision making	
Legal services for guardianship and capacity	
Financial advisor for estate planning	
